# BrainNet Viewer: A Network Visualization Tool for Human Brain Connectomics

**DOI:** 10.1371/journal.pone.0068910

**Published:** 2013-07-04

**Authors:** Mingrui Xia, Jinhui Wang, Yong He

**Affiliations:** State Key Laboratory of Cognitive Neuroscience and Learning, Beijing Normal University, Beijing, China; Semmelweis University, Hungary

## Abstract

The human brain is a complex system whose topological organization can be represented using connectomics. Recent studies have shown that human connectomes can be constructed using various neuroimaging technologies and further characterized using sophisticated analytic strategies, such as graph theory. These methods reveal the intriguing topological architectures of human brain networks in healthy populations and explore the changes throughout normal development and aging and under various pathological conditions. However, given the huge complexity of this methodology, toolboxes for graph-based network visualization are still lacking. Here, using MATLAB with a graphical user interface (GUI), we developed a graph-theoretical network visualization toolbox, called BrainNet Viewer, to illustrate human connectomes as ball-and-stick models. Within this toolbox, several combinations of defined files with connectome information can be loaded to display different combinations of brain surface, nodes and edges. In addition, display properties, such as the color and size of network elements or the layout of the figure, can be adjusted within a comprehensive but easy-to-use settings panel. Moreover, BrainNet Viewer draws the brain surface, nodes and edges in sequence and displays brain networks in multiple views, as required by the user. The figure can be manipulated with certain interaction functions to display more detailed information. Furthermore, the figures can be exported as commonly used image file formats or demonstration video for further use. BrainNet Viewer helps researchers to visualize brain networks in an easy, flexible and quick manner, and this software is freely available on the NITRC website (www.nitrc.org/projects/bnv/).

## Introduction

The human brain is naturally organized into a complex system whose topological descriptions have been represented as a structural connectome [1] of interconnected cortico-cortical axonal pathways and a functional connectome [2] of synchronized interregional neural activity. Mapping the human brain connectome and uncovering its underlying organizational principles are fundamentally important in neuroanatomy, neurodevelopment, cognitive neuroscience and neuropsychology. Recent studies have suggested that the human brain connectome can be mapped using neuroimaging data and further characterized through sophisticated analytic strategies based on graph theory [3]–[5].

Graph theoretical approaches model the human brain as collectives of nodes linked by edges, of which the nodes typically represent brain regions or voxels in neuroimaging data, while the edges are often estimated by gray matter morphological correlation [6], [7] or white matter fiber connections [8], [9] in structural data and temporal correlations [10]–[12] in functional data. Once the brain nodes and edges are extracted from the neuroimaging data, graph theoretical algorithms are further applied to measure the topological properties of the constructed networks. The application of these algorithms revealed many non-trivial topological properties of brain networks, such as small-worldness [13], modularity [14], [15], highly connected hubs [8], [16] and ‘rich-club’ configurations [17], [18]. To date, graph theoretical methods have been used to examine the relationships between human brain network properties and population attributes, such as aging [19]–[24], development [25]–[34], gender [19], [34]–[37], intelligence [34], [38], [39] and genetic [40]–[43]. Moreover, these graph-based network analysis methods have been applied to individuals with a variety of neuropsychiatric disorders [44]–[47], including Alzheimer’s disease (AD) [48]–[50], mild cognitive impairment (MCI) [51]–[53], schizophrenia [54]–[56] and epilepsy [57], [58].

Given the abstract nature of graph theoretical approaches and the huge complexity of brain networks, it is important to develop easy-to-use and efficient toolkits for graph-based network construction, analysis and/or visualization. Recently, several freely available toolkits for extracting brain network topological properties have emerged, including Brain Connectivity Toolbox (BCT) [59], eConnectome [60], Graph-Analysis Toolbox (GAT) [61], Pipeline for Analyzing braiN Diffusion imAges (PANDA) [62], NetworkX (http://networkx.lanl.gov/index.html), Brainwaver (http://cran.r-project.org/web/packages/brainwaver/index.html) and Graph-theoRETical Network Analysis toolkit (GRETNA, http://www.nitrc.org/projects/gretna/), which have greatly assisted with the investigation of the brain connectome. However, toolkits for visualizing the brain connectome as nodes and edges are still lacking.

Here, we developed a MATLAB toolbox, called BrainNet Viewer, with a Graphical User Interface (GUI), to provide a flexible and rapid visualization platform and generate figures for brain connectome studies in a user-friendly and intuitive manner. In this toolbox, the brain surface, node, edge and volume files can be defined as input containing fundamental information about brain networks, and we have designed an easy-to-use optional panel to modify the details of the network display. BrainNet Viewer automatically generates the figures as the user requires, and these figures can be saved as several common image formats for further use. Moreover, interaction functions are available to facilitate the demonstration of more detailed information.

## Materials and Methods

### Toolbox Development

#### Developing environment

**Figure 1:**
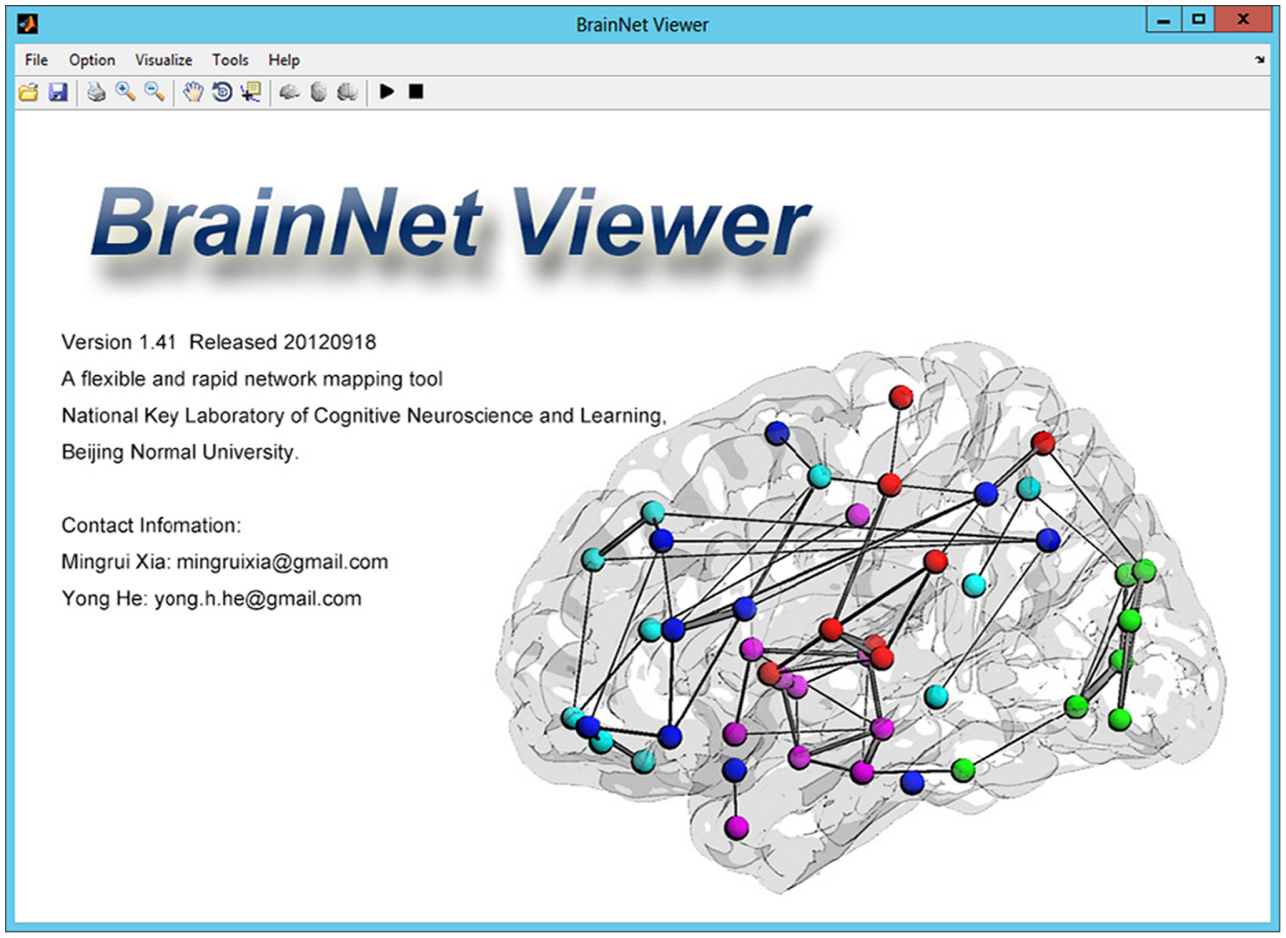
The main window of BrainNet Viewer. BrainNet Viewer is free software available on the NITRC website (www.nitrc.org/projects/bnv/), which runs with MATLAB under Windows, Linux and Mac OS, with either 32- or 64-bit systems. The latest version is 1.41, released September 18, 2012. The main window includes the menu bar, toolbar and contact information.

BrainNet Viewer was developed using MATLAB (The MathWorks Inc., Natick, MA, US) as a programming language, with a user-friendly GUI (Figure 1), under a 64-bit Windows (Microsoft Corp., Redmond, WA, US) environment. The toolbox includes functions of Statistical Parametric Mapping 8 (SPM, http://www.fil.ion.ucl.ac.uk/spm/) for loading NIfTI and Analyze format files (*.nii; *.img). This toolbox has been successfully tested under a variety of operating systems with MATLAB installed, including Windows (XP, 7, 8 and Server versions), Linux (Ubuntu and CentOS) and Mac OS in both 32- and 64-bit versions.

#### Visualization procedure

**Figure 2:**
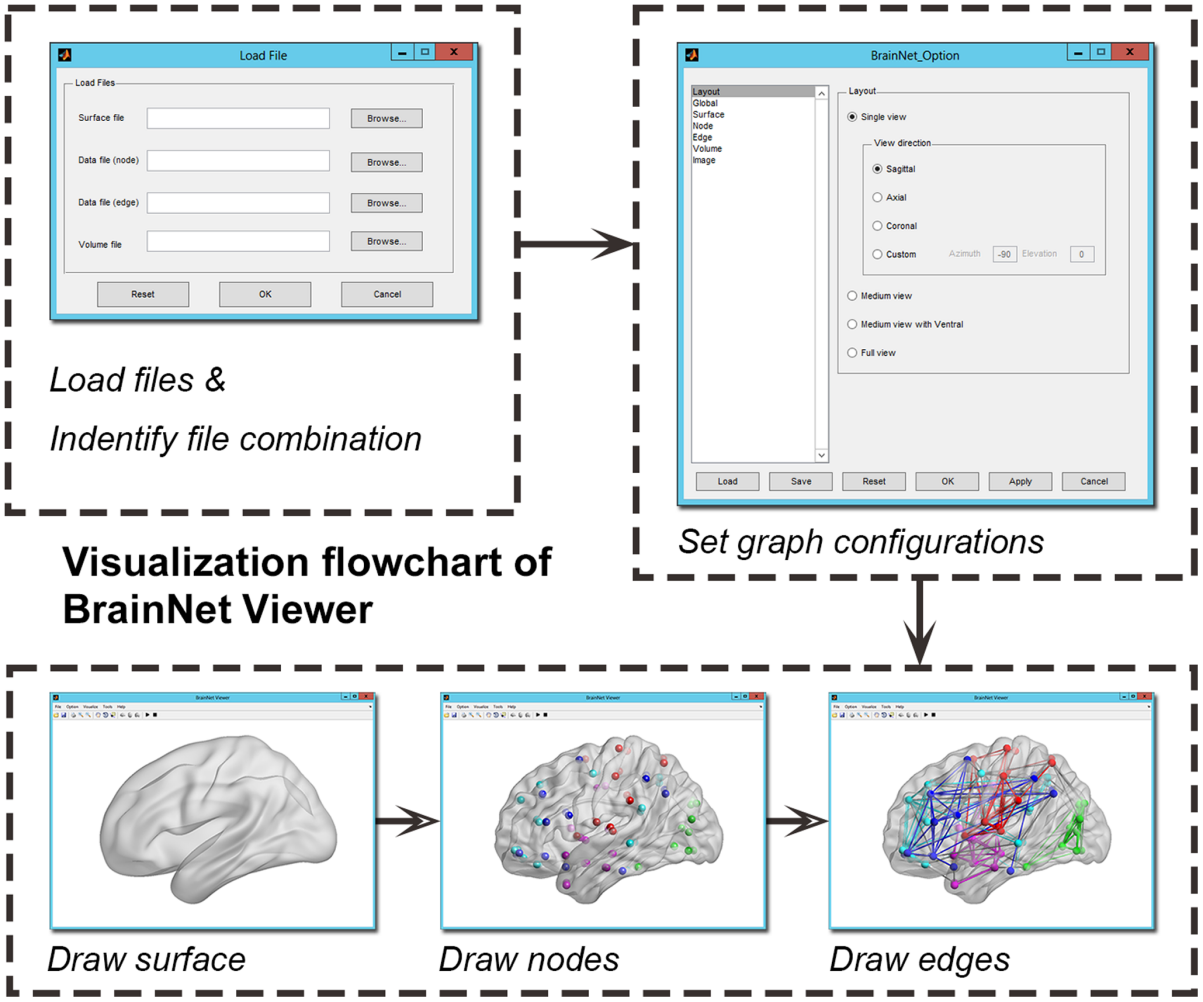
A flowchart for visualization of BrainNet Viewer. First, the combination of the files containing connectome information is loaded. Then, the configuration of the graph is adjusted in an easy-to-use option panel. Next, BrainNet Viewer draws the brain surface, nodes and edges in sequence. Finally, the figure is saved in a common image format for further use.

BrainNet Viewer was designed to visualize brain connectomes using the following procedure. First, users upload a combination of files containing connectome information, such as a brain surface, node, edge and volume files. Then, an easy-to-use options panel appears, allowing the adjustment of figure configuration parameters, such as output layout, background color, surface transparency, node color and size, edge color and size and image resolution. Subsequently, BrainNet Viewer draws the brain surface, nodes and edges (depending on the files loaded) in sequence and shows the brain network in multiple views, as required by the user. Finally, the figures are exported to common image file formats for further use (see Figure 2 for a flowchart).

#### File definition

We defined four types of import files for BrainNet Viewer, namely, brain surface, node, edge and volume files. 1) Brain surface file. The brain surface file is an ASCII text file, with the suffix ‘nv’, containing four fields: the number of vertices, the coordinates of each vertex, the number of triangle faces and the index of the vertices comprising the triangles. Currently, the ‘.pial’ file of hemisphere mesh, generated using FreeSurfer (http://surfer.nmr.mgh.harvard.edu/) [63], and the ‘.mesh’ files, generated using BrainVISA (http://brainvisa.info/) [64], are supported for direct loading and visualization. 2) Node file. The node file is an ASCII text file with the suffix ‘node’. Nodal information is arranged in 6 columns in the node file: columns 1–3 represent the x, y and z coordinates, respectively, of the nodes; column 4 represents the index for node color; column 5 represents the node size; and column 6 represents the node label. A ‘−’ symbol (no ‘’) in column 6 indicates no label for the corresponding node. The values for this file are easily arranged depending on the aspects of the network shown. For example, the modular information for the nodes can be assigned to column 4 to use color to distinguish nodes belonging to different modules. Column 5 could be set as nodal degree, centrality and T-value to emphasize nodal differences according to size. 3) Edge file. The brain edge file is an ASCII text file with the suffix ‘edge’, representing an association (e.g., correlations) matrix among the nodes, which can be weighted or binarized, and therefore, the size of the matrix must correspond to the number of nodes. Both the node and edge files can be generated or edited using text editors or spreadsheet software. 4) Volume file. BrainNet Viewer facilitates the mapping of volume data to the brain surface, which can be a functional connectivity map, gray matter density map, statistical parametric map or a brain atlas. The volume file should be in the NIfTI or Analyze format, and either a single or paired nii files are acceptable. A text file containing an n×1 vector is also acceptable, in which n equals the vertex number of the brain surface (e.g., 81,924 vertices in the International Consortium for Brain Mapping (ICBM) whole brain surface).

In BrainNet Viewer, several brain surface templates and files of example brain networks are provided. 1) The brain surface templates are primarily generated from two commonly used templates, including Ch2 (with/without cerebellum and separated hemispheres) and ICBM152 (smoothed/unsmoothed, MNI/Talaraich and separated hemispheres), which can be found in the folder ‘.\Data\SurfTemplate’. 2) The brain network files, including node and edge files, are generated from various brain parcellations, such as Automated Anatomical Labeling (AAL, 90 regions, only cerebrum) [65], Brodmann areas (82 regions) [66], Harvard-Oxford Atlas (HOA, 112 regions) [67], regions of interest (ROIs) defined by Dosenbach et al.(160 ROIs) [68], ROIs defined by Fair et al. (34 ROIs) [26] and LONI Probabilistic Brain Atlas (40 regions) [69], which are stored in the folder ‘.\Data\ExampleFiles’. Notably, the coordinates in these files are located in the MNI space, unless otherwise noted. Moreover, users are encouraged to create custom files of brain networks for visualizing specific characteristics of the brain connectome. Specifically, the customized brain surface can be extracted from anatomical data using surface reconstruction software, such as the FreeSurfer and BrainVISA. Subsequently, the number of vertices and triangle faces, vertex coordinates, and the index of the vertices in triangles of the resultant surface are stored as ASCII files with the suffix ‘.nv’ to generate a customized brain surface template. The network characteristics can be saved as ASCII files with either the ‘.node’ or ‘.edge’ suffix using text editor or Matlab commands, according to the previously described file rule definitions to generate customized network files.

#### Core codes

BrainNet Viewer manages brain network visualization in three ways: displaying graph theoretical networks as ball-and-stick models; performing volume-to-surface mapping; and constructing ROI clusters from volume files. Here, we introduce the core graphic functions in MATLAB that BrainNet Viewer uses for the visualization procedure.

The ball-and-stick model of the graph theoretical network includes three elements: brain surface, nodes and edges. 1) For the brain surface, once the vertex coordinates and triangles are loaded, the following function is used to draw the surface in a figure:

*surf_h = trisurf(tri, x, y, z);*

where *surf_h* is the handle of the object, *tri* represents the index of the vertex comprising the triangles, and *x*, *y* and *z* are the coordinates of the vertex on the surface. 2) The network nodes are represented as spheres. The sphere in a specific position (*x*, *y*, and *z*) is generated using the following codes:

*[X, Y, Z] = sphere(n);*

X = X * r+x; Y = Y * r+y; Z = Z * r +z;

*node_h = mesh(X, Y, Z);*

where *X*, *Y* and *Z* represent vertex coordinates of a unit sphere, *n* is the number of faces (*n*-by-*n*) on this sphere (in BrainNet Viewer, *n* represents the graph detail in the option panels, where 100, 50 and 20 represent high, moderate and low details, respectively), *r* is the size of the node, and *node_h* is the handle of the node. 3) Similar to nodes, cylinders are drawn in BrainNet Viewer to represent network edges. The cylinders are first generated and subsequently rotated and moved to the appropriate position in the following manner:

theta = (0:n)/n * 2 * pi; t = ones(100,1) * t;

X = t * cos(theta); Y = t * sin(theta); Z = (0:100)’/(100−1) * ones(1,n +1) * length;

*line_h = mesh(X, Y, Z);*

*rotate(line_h, axis_rot, angle_X1X2, [0 0 0]);*

where *theta* is the radian of sampling points on a circle, *n* is the number of sampling points (similar to the node sampling detail, 20, 10 and 5 represent high, moderate and low details, respectively), *t* is the radius of the cylinder, *X*, *Y* and *Z* represent vertex coordinates on the cylinder, *length* is the distance between the two nodes connected by this edge, *line_h* is the handle of this edge, and *axis_rot* and *angle_X1X2* are the vector cross product and the included angle between unit vector on the z-axis and the vector of the two nodes connected by this edge, respectively.

The procedure for volume-to-surface mapping first transfers the vertex coordinates on the brain surface to the matrix coordinates in the image file using different mapping algorithms and then assigns vertices with different values. Eight mapping algorithms are provided to determine the vertex values in BrainNet Viewer: ‘Nearest Voxel’, assign the vertex with the value of the voxel in volume that is nearest to it, suitable to display an atlas or mask; ‘Average Vertex’, assign the vertex with the value of the voxel in volume that is nearest to it, and then average the vertex across its neighbors (high time consumption); ‘Average Voxel’, assign the vertex with average value of the voxel and its neighbors in volume that is nearest to it; ‘Gaussian’, the volume first employs convolutions with a Gaussian kernel and then assigns the vertex with the value of the voxel in volume that is nearest to it; ‘Interpolated’, the coordinate of the vertex is determined in the volume space, and a trilinear interpolate method is then used across its neighbors to calculate the value; ‘Maximum Voxel’, assign the vertex with the maximum value of the voxel and its neighbors in volume that is nearest to it; ‘Minimum Voxel’, assign the vertex with the minimum value of the voxel and its neighbors in volume that is nearest to it; ‘Extremum Voxel’, assign the vertex with the extremum value of the voxel and its neighbors in volume that is nearest to it. The mapping code is similar to the surface drawing, which is represented as:

*surfmap_h = trisurf(tri, x, y, z, v);*

where *surfmap_h* is the handle of the object, *tri* represents the index of the vertices comprising the triangles, *x*, *y* and *z* are the coordinates of the vertex on the surface, and *v* represents the value of each vertex on the surface.

BrainNet Viewer also provides functions to construct ROI clusters from volume files. The voxels within the same cluster are labeled with the same index number and are fully connected. Then, the toolbox identifies and constructs the cluster from volume to surface using the following codes:

*fv = isosurface(vol);*

*roi_h = trisurf(fv.faces,fv.vertices(:,1),fv.vertices(:,2),fv.vertices(:,3));*

where *fv* represents the surface information, containing vertex coordinates (*fv.vertices*) and triangle indices (*fv.faces*), constructed from ROI clusters; *vol* is the three-dimensional matrix containing ROI clusters; and *roi_h* is the handle of the ROI object. After these objects are created, several functions controlling object properties are used to adjust the appearance of these elements in the brain network, including *EdgeColor*, *FaceAlpha*, *material*, *shading*, *lighting* and *camlight*.

### Functional Brain Network Visualization on Experimental Data

#### Subjects

To demonstrate the visualization effects of this toolbox on real data, we analyzed a published resting-state fMRI dataset. The dataset was downloaded from the 1000 Functional Connectomes Project (www.nitrc.org/projects/fcon_1000/), which is a worldwide multi-site project with fMRI data sharing for the imaging community. The resting-state images were acquired from 198 healthy right-handed volunteers (males, 76; females, 122; age, 18 - 26 years) at the scanning site of Beijing Normal University. The data for one subject were removed because of an orientation error during scanning. Each participant provided written informed consent before initiating scanning. The study was approved through the Institutional Review Board of the Beijing Normal University Imaging Center for Brain Research.

#### Image acquisition

The resting-state fMRI data acquisitions were performed on a Siemens 3T scanner. For each participant, functional images were scanned using the following parameters: repetition time = 2000 ms, echo time = 30 ms, in-plane resolution = 3.125 mm×3.125 mm, slice thickness = 3 mm, number of slices = 33, section gap = 0.6 mm, flip angle = 90°, field of view = 200 mm×200 mm and time points = 225. The participants were instructed to remain awake with their eyes closed during the scanning.

#### Image pre-processing

The image pre-processing was conducted using DPARSF [70] and SPM5 (www.fil.ion.ucl.ac.uk/spm/). The first 10 volumes of each participant were removed to for the adaptation of the participants to the scanning noise. The following pre-processing steps included slice timing, realignment, spatial normalizing to the standard EPI template in MNI space and resampling to an isotropic 3-mm voxel size, spatially smoothing with a 4-mm FWHM kernel, detrending and band-pass filtering (0.01 - 0.08 Hz). Furthermore, we regressed out the white matter (WM), cerebrospinal fluid (CSF), global signals, and head-motion profiles to reduce the effect of these nuisance signals.

#### Network construction

We constructed the functional brain networks for each individual using two methods, differentiated according to the node definition, as either a region- or voxel-based network. 1) The region-based networks were constructed following the following manner. First, the AAL atlas was used to parcellate the entire brain into 90 regions (regions in cerebellum were excluded), which were considered as nodes in the network. Then, the mean time courses were extracted from each region and used to obtain a 90×90 correlation matrix of Pearson’s correlation coefficients between all possible connections of node pairs. 2) The voxel-based networks were constructed by directly considering GM voxels as nodes. The Pearson’s correlation coefficients were then computed between the time courses of all pairs of voxels to generate a ∼50,000×50,000 correlation matrix. All correlation matrices were transferred into z-score matrices using Fisher’s r-to-z transformation to improve normality.

#### Network analysis

We analyzed the region-based networks at the group level. First, we performed one sample t-tests for all possible connections across all subjects, and the t-values were considered as the strength of connections. Then, a Bonferroni-corrected significance level of P<0.05 was used to remove the non-significant connections and obtain a group-level weighted functional matrix (network). Notably, the negative correlations were excluded because of biological ambiguity. Subsequently, a modular detection algorithm [14] was applied to the resultant weighted network to identify functional modules. Finally, we calculated the functional connectivity strength (FCS; i.e., nodal strength) for each node by summing the connections (t-score) linked, and the resultant FCS values were further normalized to standard Z-scores (the minus mean and divided by the standard deviation (SD)). The nodes with a Z-score higher than 1 were identified as network hubs.

For the voxel-based network analysis, the FCS in the brain functional network of each subject was calculated. The FCS of a voxel was computed as the sum of the connections (z-score) between the given voxel and all other voxels. We conservatively restricted the analysis to positive correlations above a threshold of r = 0.2. The FCS maps were averaged across subjects, and the resultant mean FCS map was further normalized (the minus mean and divided by the SD) to exhibit the hub distribution of brain functional networks on a group level. Given a high computational load, we did not analyze the other network properties, such as modularity.

## Results

### Toolbox Development

#### Download and installation

The BrainNet Viewer package is available as a free download from the NITRC website (www.nitrc.org/projects/bnv/), and this software is also listed among the SPM extensions (www.fil.ion.ucl.ac.uk/spm/ext/#BrainNetViewer). The BrainNet Viewer has been downloaded over 4,400 times from the NITRC website since it was released on July 7, 2011. The installation of BrainNet Viewer is similar to most MATLAB toolboxes. To run this package, open MATLAB, add the BrainNet Viewer folder in the MATLAB search path, and type ‘BrainNet’ in the command window of MATLAB. In addition, a user-friendly manual is also available within the package, providing a detailed guide for using BrainNet Viewer.

#### Combinations of files

**Figure 3:**
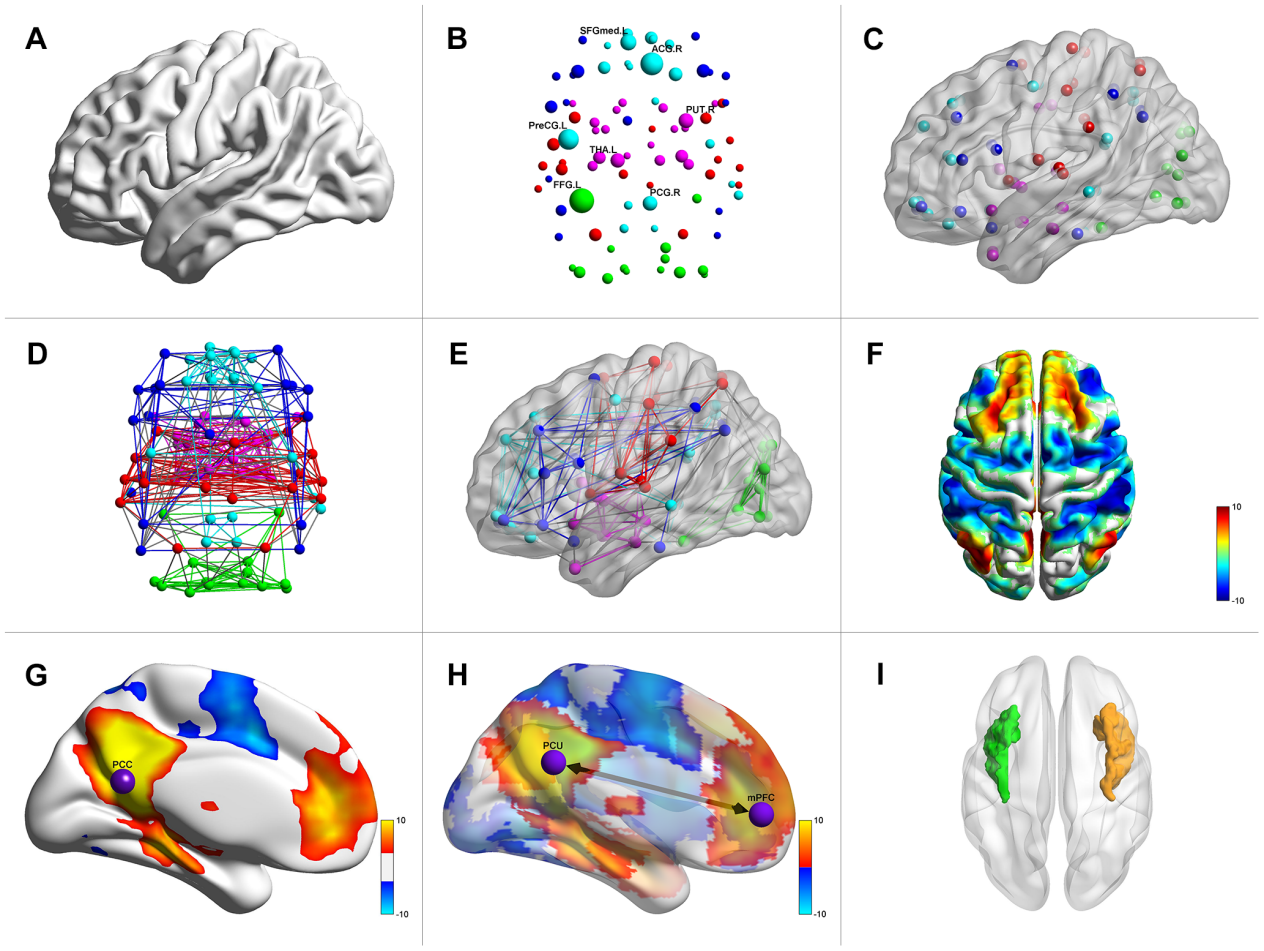
Pictures generated from different file combinations. The file combinations that BrainNet Viewer accepts include the following: (A) brain surface only; (B) nodes only; (C) brain surface with nodes; (D) nodes and edges; (E) brain surface with nodes and edges; (F) brain surface and volume files for volume-to-surface mapping; (G) brain surface, nodes and volume files for volume-to-surface mapping with nodes; (H) brain surface, nodes, edges and volume files for volume-to-surface mapping with nodes and edges; and (I) brain surface and volume files for regions of interest construction.

Although four types of input files are defined for BrainNet Viewer, it is not necessary to load all files types at one time. Instead, several combinations are acceptable, and different combinations will generate different network pictures. These combinations include 1) brain surface file only; 2) node file only; 3) brain surface and node files; 4) node and edge files; 5) brain surface, node and edge files; 6) brain surface and volume files for volume-to-surface mapping or ROI cluster drawing; 7) brain surface, node and volume files; and 8) brain surface, node, edge and volume files. Figure 3 shows the sample images generated using these different combinations.

#### Option setting

**Figure 4:**
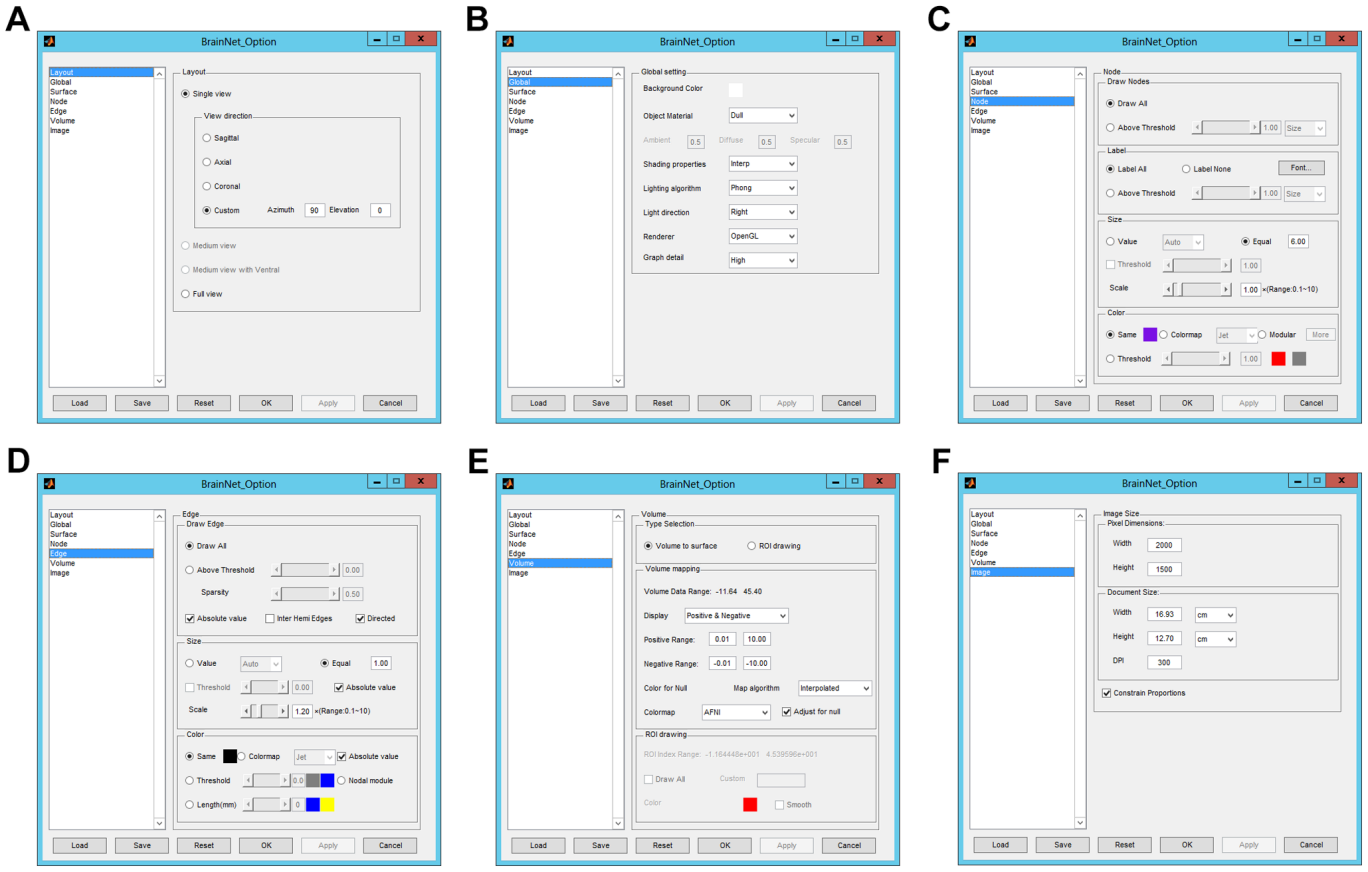
The option panel and its subpanels in BrainNet Viewer. (A) The layout panel is adopted to set the output view of the brain model. (B) The global panel is responsible for the adjustment of the display properties. (C) The node panel is developed to control node label, node size and node color. (D) The edge panel is employed to control edge extraction, edge size and edge color. (E) The volume panel is used for setting volume-to-surface mapping and regions of interest construction. (F) The image panel is applied for determining the parameters of the output image.

We developed an option panel (Figure 4) in BrainNet Viewer for adjusting the details of the figure intuitively and easily. The option panel is divided into seven subpanels, corresponding to different aspects of the figure, including layout, global, surface, node, edge, volume and image, switched from the list box on the left of the panel. In addition, the configuration in this panel can be saved as a.mat file and recalled at next use or in a command line (see Command line section). These panels are briefly defined using the following description.

**Figure 5:**
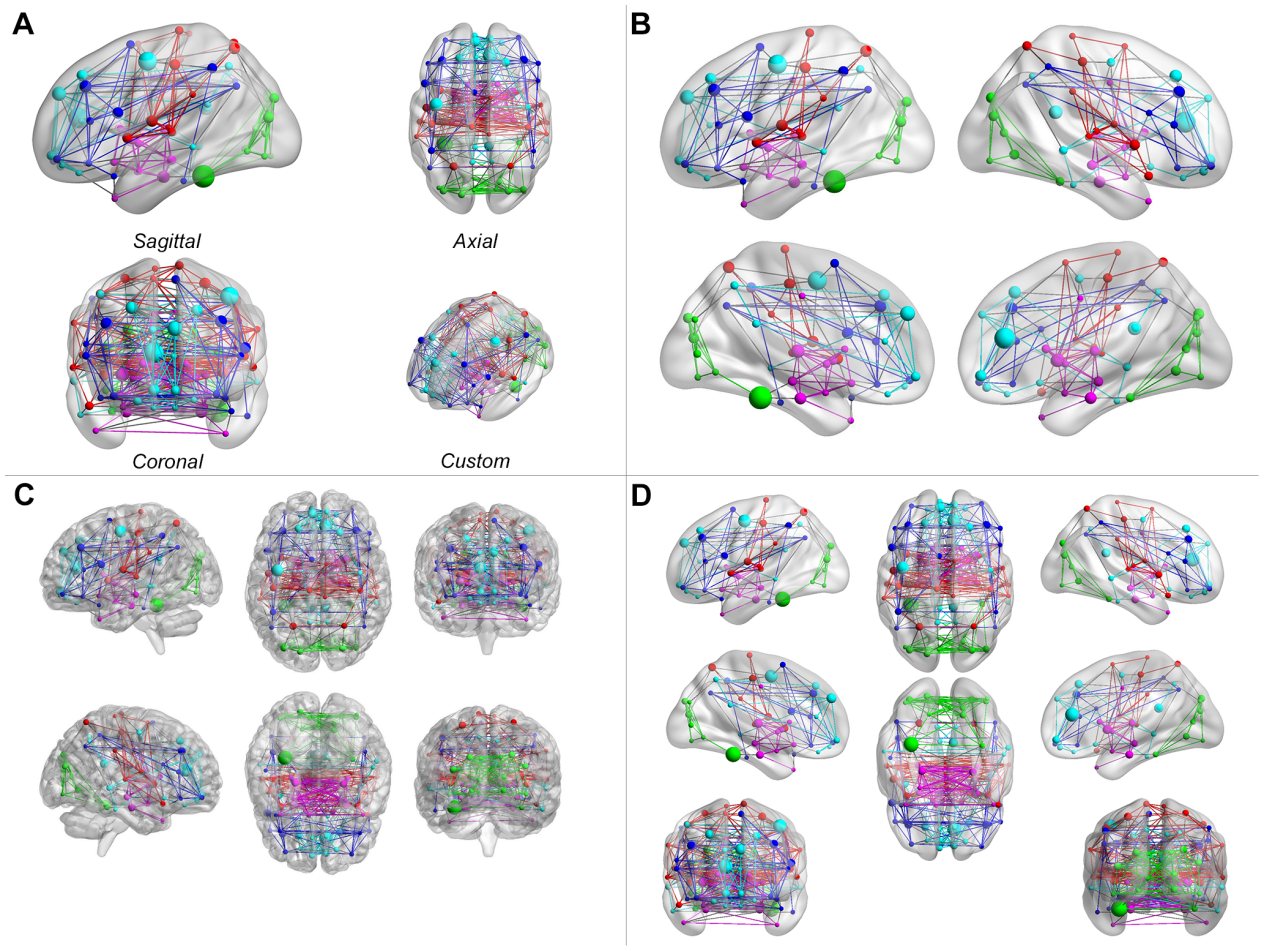
Different layouts of brain models. (A) The single view shows a single brain model in the figure as one of the three standard (sagittal, axial or coronal) views or a custom camera view. (B) The medium view shows the lateral and medial sides of each hemisphere in the figure. (C and D) The full view shows all sides of the brain surface. According to whether the brain surface file can be divided into two hemispheres, this mode displays brain models in two ways: (C) if not divisible, the left, right, dorsal, ventral, anterior and posterior sides are displayed separately; (D) otherwise, the lateral and medial sides of each hemisphere, and the dorsal and ventral sides and the anterior and posterior sides of the entire brain are shown.

**Figure 6:**
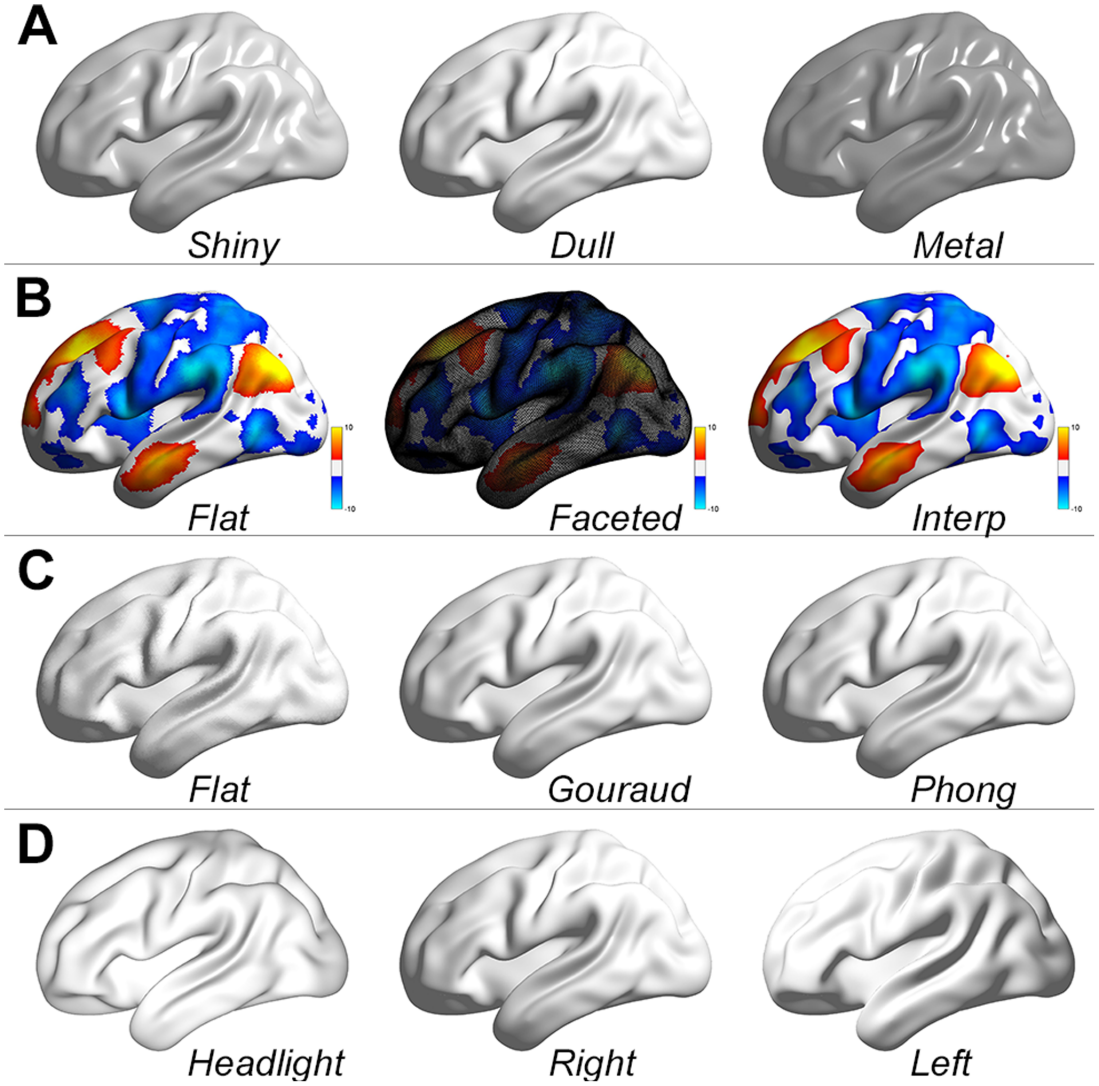
Demonstration of object properties. (A) Material property controls the reflectance properties of the surfaces, including choices of shiny, dull and metal. (B) The shading property controls the color shading for the surface, including choices of flat, faceted and interp. (C) The lighting property changes the lighting algorithm from flat, gouraud and phong. (D) The light direction determines where the light comes from, i.e., headlight, right or left.

**Figure 7:**
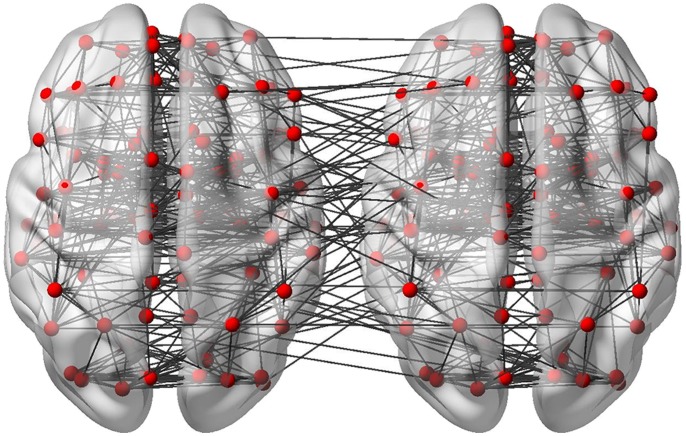
Interactions between two brains. BrainNet Viewer illustrates the interactions between two brains, as demonstrated here. The nodes used in this figure were extracted from the AAL90 template, while the connection between each pair of two nodes was randomly generated.

1. Layout panel. The layout panel (Figure 4A) is primarily responsible for setting the output view of the brain model, in which three types of views are provided: the single view shows only one brain model in the figure (Figure 5A); the medium view shows the lateral and medial sides of each hemisphere in the figure (Figure 5B); and the full view shows all sides of the brain surface. Depending on whether the inputted brain surface can be divided into two hemispheres, the layout panel shows brain models in two ways: if not dividable, the left, right, dorsal, ventral, anterior and posterior sides are displayed separately (Figure 5C); otherwise, the lateral and medial sides of each hemisphere, the dorsal and ventral sides, and the anterior and posterior sides of the entire brain are shown (Figure 5D).
2. Global panel. The global panel (Figure 4B) provides several different choices for the adjustment of the global figure, particularly the display properties of these objects. Here, users can change the color of the background, select material for the objects (Figure 6A), change shading properties (Figure 6B), select the lighting algorithm (Figure 6C), determine where the light comes from (Figure 6D), change the rendering method and set the graph details (e.g., set the number of sampling points for nodes and edges).
3. Surface panel. The surface panel is available for adjusting the properties of the brain surface. The surface panel is simple, with only three options: the surface color, the opacity of the surface and a switch for displaying the interaction of two brains in one figure (Figure 7).
4. Nodal panel. The node panel (Figure 4C) is developed with four zones to select node drawing, set labels, and adjust the node size and color, respectively. All settings are dependent on the nodal information in the nodal file. Users can draw all nodes contained in the nodal file or select a subset by setting a threshold for the value of column 4 or column 5 in the nodal file. The nodes with higher values than the threshold will be shown. The labels for these nodes can be added using the text of column 6 in the node file, and the font type and size can be selected. BrainNet Viewer provides three ways to adjust the node size: automatically arrange the sizes of the nodes to a proper range, according to nodal size in the node file; use the original value in the node file; or set all nodes to an equal size defined in the panel ignoring the size value in the file. Node color can be adjusted in four ways: using the same color for all nodes, ignoring the color index in the file; using a color map to display the values of the nodes from low to high, corresponding to the color index in the node file; assigning distinct colors for nodes labeled with different modular indices in column 4 of the node file; or binarizing the color to a given threshold.
5. Edge panel. The edge panel (Figure 4D) is similar to the node panel, with three parts that separately control edge extraction, edge size and edge color. The edges are extracted from the association matrix contained in the edge file by setting a threshold of either a real value or sparsity (i.e., density or cost). BrainNet Viewer can also extract edges using an absolute value in the matrix or only edges that travel across two hemispheres. In addition, the asymmetric matrix can be used to draw edges with direction. There are three ways to adjust the radius of edges: automatically arrange the sizes of edges to a proper range according to the values in the association matrix in the edge file; use the original value of the association matrix; or set all edges to an equal radius defined in the panel, ignoring the size values in the file. BrainNet Viewer provides five ways to set edge color: adopt the same color for all edges; use a color map to render edges by their values from low to high; binarize the color by a given threshold for edge value; binarize the color by a given threshold of Euclidean distance between two nodes connected by this edge; or assign edge color according to the colors of the nodes that it links.
6. Volume panel. The volume panel (Figure 4E) is set to control the volume-to-surface mapping and draw ROI clusters with brain surface. In the volume-to-surface mapping section, the users perform mapping with positive, negative or both positive and negative values in the volume file. We provide 24 types of colorbars, including those most commonly used in research, such as jet, hsv, hot, cold, winter and summer. In addition, a custom colorbar can be generated using an n×3 matrix. Eight mapping algorithms are available, as described in the Methods section, providing various effects for mapping. In the ROI drawing section, users select the index number and set the color of the ROI clusters that need to be reconstructed and drawn.
7. Image panel. In the image panel (Figure 4F), the configurations are related to the size and resolution of the output images. The width and height of the image can be adjusted in pixel dimensions for screen display or in real units (centimeter or inch) for document use. The resolution of the output image can also be modified in dots per inch (DPI).

#### User interaction

**Figure 8:**
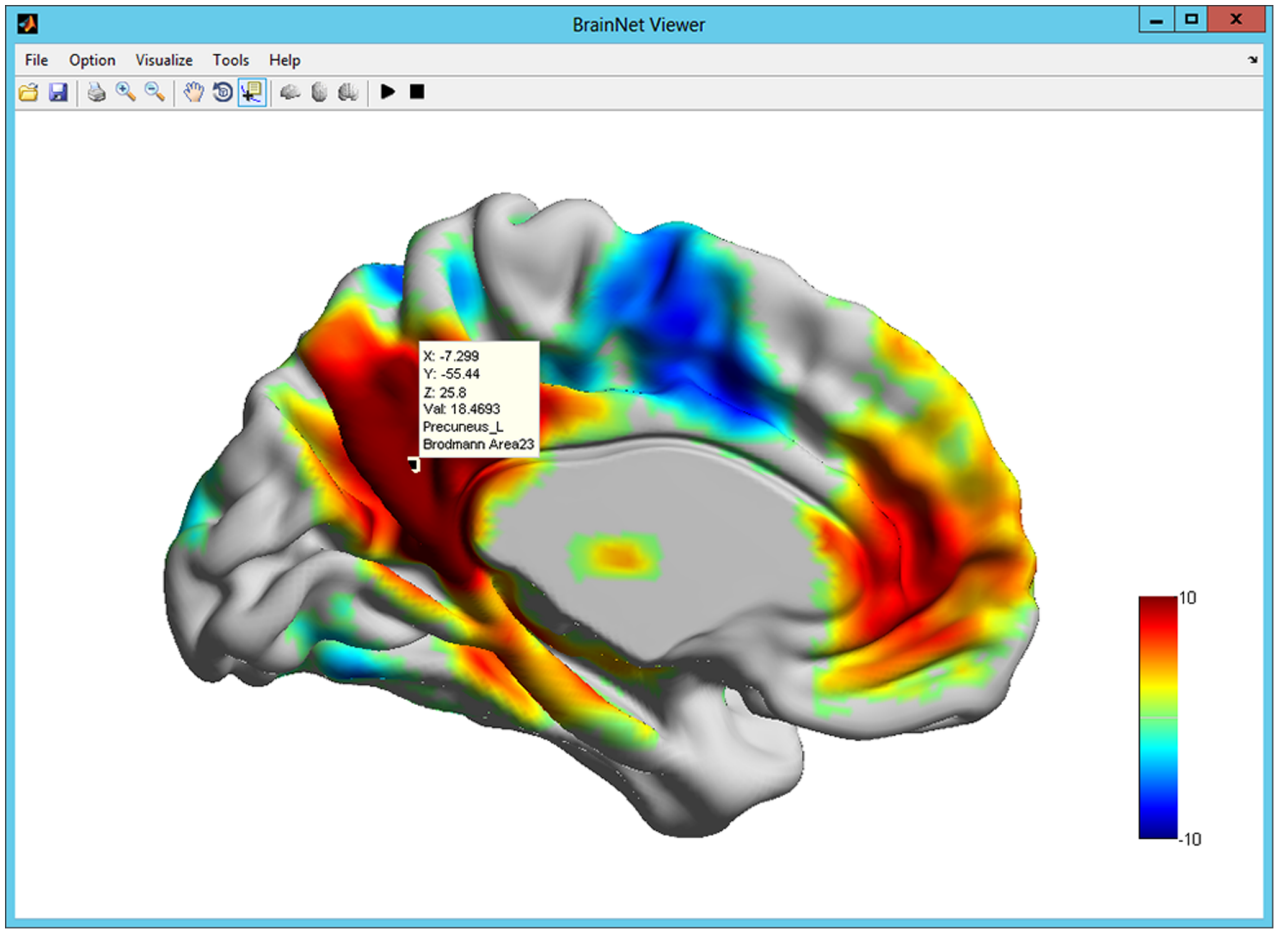
The data tip displayed using the ‘Data Cursor’ function. The ‘Data Cursor’ function on the toolbar in BrainNet Viewer is used to interactively obtain information about the vertex on the brain surface. When this function is enabled, clicking anywhere on the brain surface will generate a data tip with the coordinates and values for the selected vertex and the AAL brain region and Brodmann Area where the vertex belongs. The vertex selected in this figure shows an MNI coordinate of x = −7.3, y = −55.4 and z = 25.8, with a statistic T value of 18.47. Furthermore, this vertex belongs to the left precuneus in the AAL template and the Brodmann region 23.

On the toolbar for the main window, some interactional operation functions, including zoom in, zoom out, move, rotate, data cursor, standard views and demonstration, were developed. The zoom in and zoom out functions help to observe the local or global status of the brain network. With “move” and “rotate” functions, users can move or change the view of the brain model by dragging with the mouse. The data cursor function displays the coordinates and value of the vertex on the surface, and it also provides the corresponding brain region labels in terms of AAL and Brodmann atlases (Figure 8). Shortcuts for three standard views, sagittal, axial and coronal, are available to quickly observe networks from different standard views. The demonstration function makes the brain model rotate clockwise until terminated by the user.

#### Output

The brain connectome figures can be saved as several common image formats, including TIFF, BMP, EPS, JPEG and PNG. Moreover, BrainNet Viewer can save the brain networks as videos, generating a 12 second long, 30 FPS, 735×534, AVI file, in which the brain network model rotates clockwise in a circle at one degree per frame. Generating these videos takes approximately 10 minutes (for an example, see www.nitrc.org/docman/view.php/504/1023/Demo%20Video%20of%20Brain%20Network%20(14M)).

#### Command line

Considering the growing requirements for batched brain connectome figure mapping, such as dynamic brain functional connectomes, the functionality to generate brain network figures in the command line is provided. The function is called according to the following command line:

*BrainNet_MapCfg(filename1, filename2…);*

where the variables of filenames can be any one of the brain surface, node, edge and volume files. Once the files are loaded, BrainNet Viewer draws the graphs with default configurations. For instance, a command line of

*BrainNet_MapCfg(‘BrainMesh_ICBM152.nv’, ‘Node_AAL90.node’);*

will draw the brain surface of ‘BrainMesh_ICBM152.nv’ and nodes in ‘Node_AAL90.node’ files using default settings.

A pre-saved configuration file can also be included in this command line. For example, the command line.

*BrainNet_MapCfg(‘BrainMesh_ICBM152_smoothed.nv’, ‘OneSample_T.nii’, ‘Cfg.mat’);*

would map the volume ‘OneSample_T.nii’ onto brain surface ‘BrainMesh_ICBM152_smoothed.nv’ using the settings pre-saved in the ‘Cfg.mat’ file.

The command line also supports exporting the brain network figure as image file. The names of the required image files are added to the command line:

*BrainNet_MapCfg(‘Node_AAL90.node’,’Edge_AAL90_Binary.edge’, ‘Net.jpg’);*

Using this command, BrainNet Viewer draws a network in which the node information is obtained from ‘Node_AAL90.node’ and the edge information is obtained from ‘Edge_AAL90_Binary.edge’ using default settings, and this figure will be saved as a JPEG image as ‘Net.jpg’. The order of these inputted filenames is exchangeable, and the combinations of files are similar to the GUI version.

### Functional Brain Network Visualization on Experimental Data

#### Biological findings and network visualization

**Figure 9:**
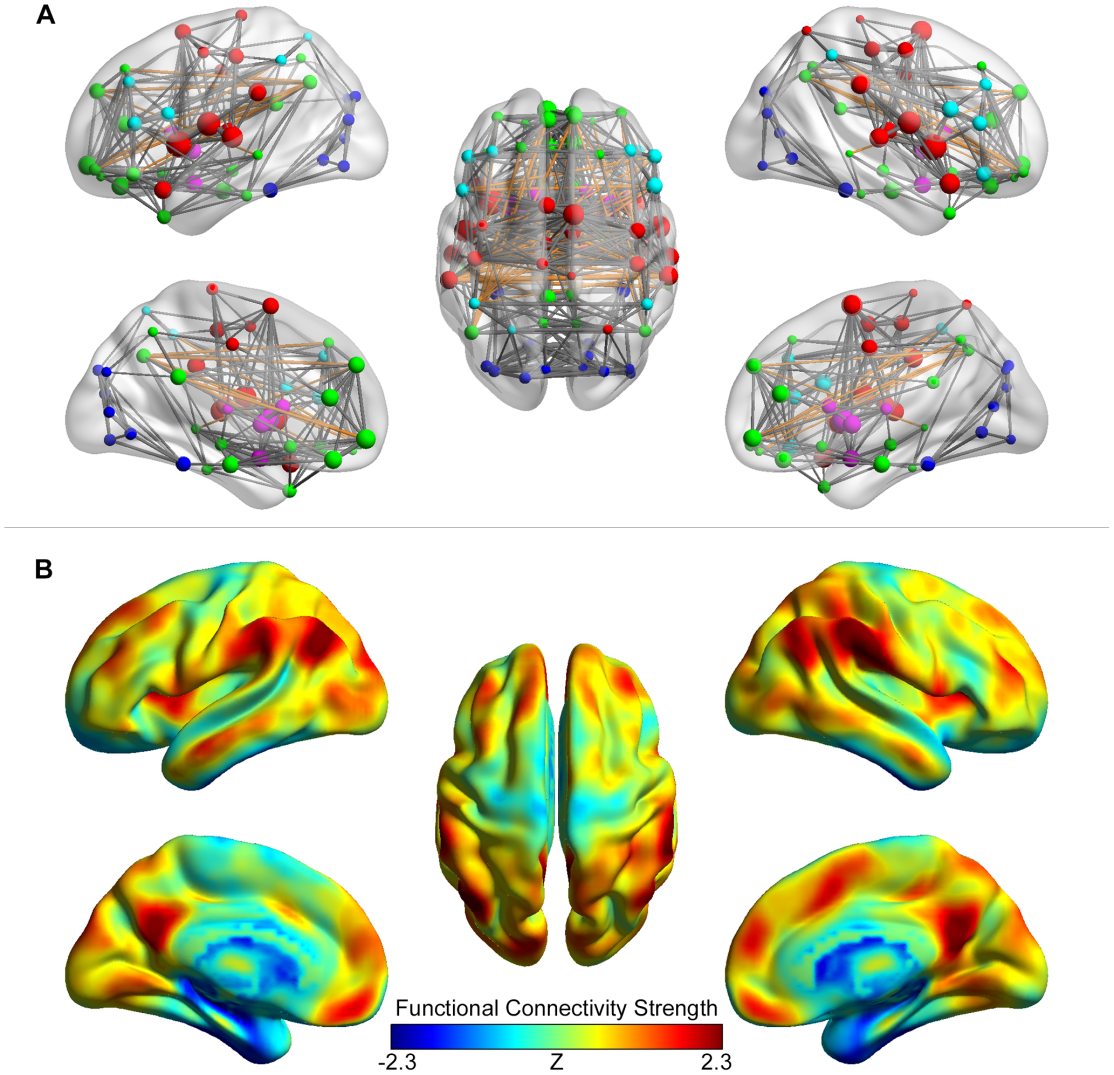
Visualization of functional brain networks. (A) The region-based network is shown as a ball-and-stick model. The nodes are brain regions in the AAL atlas and their coordinates, sizes and colors represent the centroids, nodal strengths and modules of the regions, respectively. The edges represent the connections between different brain regions (top 15% are displayed). The functional connectivity strengths are presented as the radius of the edges, and long-distance connections (>90 mm) are colored in orange. (B) The voxel-based network is shown using volume-to-surface mapping. The values of the vertices on the surface indicate the normalized functional connectivity strength of the voxels in the volume data. Several hub regions with high connectivity strength are rendered in warm colors.

Figure 9A illustrates the region-based functional network as ball-and-stick models. The coordinates of the nodes were centroids of the brain regions in the AAL atlas. The sizes of the nodes were assigned with a value for the nodal strength. Several hub regions were identified, including the bilateral Rolandic operculum, bilateral superior temporal gyrus, right supplementary motor area, right temporal pole, right supramarginal gyrus, left medial orbital superior frontal gyrus, bilateral insula and bilateral putamen, which were primarily located at the association and subcortical regions. In addition, five functional modules were identified in this network, and their nodes were rendered using different colors: the module in green comprises the regions in the default-mode network; the module in cyan comprises the regions predominantly involved in the attention and execution control; the module in red comprises the region of the sensorimotor cortex; the module in blue comprises the regions of the visual cortex; and the module in magenta comprises the regions of the subcortical nuclei.

The top 15% connections in strength were displayed as edges in the network. The radius of an edge represented the weight of the connection between the two linked nodes. Through visual inspection, the connections within each module were denser than the connections between two modules. The long-distance connections (>90 mm) were colored in orange. Intriguingly, the long-distance connections were primarily linked the homologous regions in two hemispheres and the anterior and posterior portions of the default-mode network within each hemisphere.

Figure 9B illustrates the FCS map of the voxel-based functional network using volume-to-surface mapping. Hub regions, exhibiting high FCS values, were predominantly observed in the default-mode network, including the medial prefrontal cortex/ventral anterior cingulate cortex, dorsal prefrontal cortex, precuneus/posterior cingulate cortex and inferior parietal lobule. Other regions included the visual cortex and the insula. These results were obviously different from those in the region-based network, suggesting that the hub distribution of the brain functional networks was dependent on the spatial scales.

## Discussion

We developed BrainNet Viewer as a free software for visualizing macro-scale brain networks (or connectomics), which achieved the following major functions: 1) display brain networks in multi-views; 2) display combinations of brain surface, nodes and edges; 3) adjust properties of network elements (i.e., nodes and edges); 4) map the volume image to brain surface; 5) support various types of image format exporting and video making; and 6) provide interactive operations, such as zoom and rotate. In addition, we constructed functional brain networks from a public dataset and further analyzed and visualized the topological properties of the resultant brain networks.

BrainNet Viewer visualizes the topological properties of brain networks constructed through region-based or voxel-based methods, as illustrated in Figure 9. For the region-based network, this toolbox displays the nodes and edges in their positions corresponding to the brain regions and adjusts their color and size according to required properties. Notably, we selected the AAL atlas to build a region-based network and visualize its topological architecture using this software. Given that the AAL atlas is one of the most widely used templates in human brain connectome studies, the use of this atlas is representative for connectome visualization. Notably, other atlases are generated from anatomical [66], [69] or functional [26], [67], [68] parcellations. These different parcellations reflect the different organizational information in the brain, and BrainNet Viewer provides different parcellation choices for network node definition. For the voxel-based network, we used volume-to-surface mapping to demonstrate nodal properties. The visualization results distinguish the topological differences over the entire brain surface. Moreover, several recent R-fMRI studies have explored the temporal dynamics of the functional brain connectome [71]–[74]. In these studies, brain networks are often constructed at individual or a period of time points, requiring a series of network figures. With BrainNet Viewer, researchers easily generate batched figures or network videos by writing loop codes. Compared with traditional visualization methods, which illustrate the network as a mosaic-like matrix or a dot-and-line plot in a plane, BrainNet Viewer generates a three-dimensional display of the networks, intuitively provides much more anatomical information for the brain and exhibits diversity using both graph-based network demonstration and volume-to-surface mapping [75]. These advantages make BrainNet Viewer a promising visualization platform for brain connectome studies and might inspire new ideas for understanding the construction principles of brain networks.

With the advent of brain connectome studies, a number of toolboxes were developed to construct and analyze macro-scale brain networks, including PANDA, BCT, GAT, GRETNA, Brainwaver and eConnectome. These toolboxes provide measurements of brain connectome features but lack the visualization options necessary to demonstrate biological findings. NetworkX and Pajek (http://vlado.fmf.uni-lj.si/pub/networks/pajek/) are two popular network visualization toolboxes to demonstrate network topological properties using dots and lines. These programs are suitable for displaying networks constructed from different fields, including the genome, society, traffic and telecom, however, ignoring the specific anatomical information of the brain science. Caret (http://brainvis.wustl.edu/wiki/index.php/Caret:About) is a widely used toolbox for the visualization and analysis of the brain cortex, providing an abundant operation at the brain surface level, but lacking the demonstration for the topology of graph-based networks. Compared with these connectome toolboxes, BrainNet Viewer has an advantage of visualizing the topology of the macro-scale brain networks with detailed, predefined brain anatomical or functional information. Another brain connectome visualization toolkit, Connectome Viewer [76], offers comparable visualization functions, through a python-based, not Matlab-based, toolbox. Considering that most of the graph-based brain network analysis toolboxes were developed in a Matlab environment, our toolbox exhibits better compatibility and usability, such as software for the interfaced and batched generation of images. Notably, there are also simulation and visualization toolboxes for modeling neuronal networks at a micro-scale, including neuroConstruct (www.neuroconstruct.org/) [77], PyNN (neuralensemble.org/PyNN/) [78] and CPT (Connectivity Pattern Tables) [79]. Because these toolboxes focus on the mechanism of neuronal activities underlying macro-scale brain networks, we can hardly compare these software programs with BrainNet Viewer. The toolboxes described above facilitate brain network studies from different aspects, and their main features are summarized in Table 1.

**Table 1:** Summary of neuroscience networks tools.

Name	Category	Feature	Environment	Specific file format	Website
*BrainNet Viewer*	Network visualization	3D graph-based brain network demonstration with nodes and edges; 3D brain surface view	Matlab-based; Windows & Linux	.nv;.node;.edge	www.nitrc.org/projects/bnv/
*Connectome Viewer*	Network visualization	3D graph-based brain network demonstration with nodes and edges; 3D brain surface view	Python-based; Linux only	.cff	http://cmtk.org/viewer/
*Caret*	Network visualization	3D surface view and nodes	C++-based; Windows & Linux	.spec	http://brainvis.wustl.edu/wiki/index.php/Caret:About
*NetworkX*	Network calculation & visualization	Graph-based network analysis; 2D demonstration with dots and lines	Python-based; Windows & Linux	.net	http://networkx.github.io/
*Pajek*	Network visualization	Graph-based network analysis; 2D demonstration with dots and lines	Delphi-based; Windows only	.pjk	http://vlado.fmf.uni-lj.si/pub/networks/pajek/
*PANDA*	Network construction	Network construction from dMRI data	Matlab-based; Linux only	NA	www.nitrc.org/projects/panda/
*GRETNA*	Network construction & calculation	Network construction from Resting-state fMRI data; graph-based network analysis	Matlab-based; Windows & Linux	NA	www.nitrc.org/projects/gretna/
*BCT*	Network calculation	Graph-based network analysis	Matlab-based; Windows & Linux	NA	https://sites.google.com/site/bctnet/
*GAT*	Network construction & calculation	Network construction from MRI data; graph-based network analysis	Matlab-based; Windows & Linux	NA	http://nnl.stanford.edu/tools.html
*Brainwaver*	Network construction & calculation	Network construction from Resting-state fMRI data (wavelet); graph-based network analysis	R-based; Windows & Linux	NA	http://cran.r-project.org/web/packages/brainwaver/
*eConnectome*	Connectivity calculation	EEG data preprocessing; connectivity analyze	Matlab-based; Windows & Linux	NA	http://econnectome.umn.edu/
*neuroConstruct*	Neuronal network modeling & visualization	Neuronal network analyze; 3D demonstration with neuronal morphology	Java-based; Windows & Linux	NA	www.neuroconstruct.org/
*PyNN*	Neuronal network modeling	Neuronal network simulation	Python-based; Linux only	NA	neuralensemble.org/PyNN/
*CPT*	Neuronal network visualization	2D demonstration of neuronal network in matrix view	Algorithm only	NA	NA

Abbreviations: PANDA, Pipeline for Analyzing braiN Diffusion imAges; BCT, Brain Connectivity Toolbox; GAT, Graph-Analysis Toolbox; GRETNA, Graph-theoRETical Network Analysis toolkit; Caret, Computerized Anatomical Reconstruction and Editing Toolkit; CPT, Connectivity Pattern Tables; NA, not available.

Since the BrainNet Viewer was released on the NITRC and SPM websites, many researchers have adopted this toolbox to visualize the characteristics and divergences of brain networks for connectome-based methodological studies [80], [81] and under healthy and diseased conditions, such as age [34], gender [34], [36], intelligence [34], AD [53], [82], [83], MCI [51], [53], [84], [85], depression [84], [86]–[88], epilepsy [58], [89], [90] and addiction [91]. In addition, some of these figures were selected as cover images for several high-level neuroscience journals [51], [84], [87], [92], [93]. Moreover, several network analysis toolboxes provide interfaces with network visualization software. For example, BCT generates files for Pajek and Connectome Viewer. Notably, several brain connectome toolboxes, such as GAT [61], GRETNA and RESting-state fMRI data analysis Toolkit (REST, www.restfmri.net), provide user-friendly interfaces to directly call the functions of the BrainNet Viewer. The connections between BrainNet Viewer and these network analysis toolboxes help researchers easily visualize and assess their results.

Although BrainNet Viewer addresses the challenges in the visualization of the brain connectome, a few methodological considerations and directions require future study. As with other toolboxes developed in the MATLAB environment, BrainNet Viewer has advantages in development and maintainability. However, the common problems of high memory consumption and slow loop execution for MATLAB programs exist in BrainNet Viewer, similar to other MATLAB packages. Notably, BrainNet Viewer fluently manages networks constructed using hundreds of nodes and lower sparsity of edges. When the number of nodes increases to the level of thousands, the rendering speed becomes slow, and the memory consumption increases quickly. The ‘out of memory’ error sometimes occurs on 32-bit operating systems when dealing with a large network (e.g., 50,000). There might be two ways to solve this problem. On one hand, the codes of the toolbox could be further optimized to minimize the memory consumption. However, such an optimization is still limited under the MATLAB framework. On the other hand, translating the source code to a more efficient programming language, such as Python or C, might be a more efficient solution. With the rapidly increasing numbers of brain connectome studies, requirements for different manners of visualization are also mushrooming. For instance, in BrainNet Viewer, the brain connectome is treated as a brain surface using a ball-and-stick model; however, in reality, the brain regions and interregional connections are typically irregular objects and long thin fibers, instead of simple balls and sticks. Showing the brain connectome in both realistic and abstract ways might enhance our understanding of its underlying principles. Furthermore, we will improve the current version by including more functions, such as automatic placement of the nodal labels without overlapping, statistical analysis, slice image display and improvements to the user experience.
